# Eating Behaviors, Dietary Patterns and Diet Quality in Newly Diagnosed, Treatment-Naïve Children with ADHD: An Age- and Sex-Matched Case–Control Study

**DOI:** 10.3390/nu18193176

**Published:** 2026-09-26

**Authors:** Nilüfer Şen, Nilüfer Acar Tek

**Affiliations:** 1 Program of Nutrition and Dietetics, Graduate School of Health Sciences, Gazi University, Ankara 06540, Türkiye; 2 Department of Nutrition and Dietetics, Faculty of Health Sciences, Gazi University, Ankara 06490, Türkiye

**Keywords:** attention-deficit/hyperactivity disorder (ADHD), eating behavior, dietary patterns, MIND diet, highly processed foods, children

## Abstract

**Background/Objectives**: Evidence on eating behaviors and dietary characteristics in children with ADHD is limited by clinical heterogeneity and narrow assessment approaches. This exploratory matched case–control study compared eating behaviors, diet quality, highly processed food consumption, and empirically derived dietary patterns between newly diagnosed, treatment-naïve children with ADHD and age- and sex-matched healthy controls. **Methods**: The study included 64 children with ADHD without major comorbidities and 64 healthy controls individually matched by sex and age (±6 months). Eating behaviors were assessed using the Children’s Eating Behaviour Questionnaire and Behavioral Pediatrics Feeding Assessment Scale. Dietary intake was assessed using a food frequency questionnaire; diet quality, highly processed food consumption, and dietary patterns were evaluated using the MIND Diet Index, the Screening Questionnaire of Highly Processed Food Consumption (sQ-HPF), and principal component analysis, respectively. Each exposure was examined separately using conditional logistic regression, with maternal education included in the primary adjusted models and Holm correction applied across exposure analyses. **Results:** Children with ADHD had higher desire to drink and sQ-HPF scores and lower MIND Diet Index and healthy dietary pattern scores than matched controls. In the primary adjusted models, higher desire to drink scores (OR = 1.88, 95% CI: 1.37–2.58) and sQ-HPF scores (OR = 2.44, 95% CI: 1.61–3.71) were associated with higher odds of ADHD, whereas higher MIND Diet Index scores were associated with lower odds (OR = 0.77, 95% CI: 0.66–0.89); these associations remained significant after Holm correction. The healthy dietary pattern association did not remain significant after correction. **Conclusions**: In this exploratory matched study, children with ADHD had higher desire to drink and sQ-HPF scores and lower MIND Diet Index scores; however, the observational design precludes temporal or causal inference, and larger independent prospective studies are needed.

## 1. Introduction

Attention-deficit/hyperactivity disorder (ADHD) is one of the most common neurodevelopmental disorders of childhood and is characterized by developmentally inappropriate and persistent symptoms of inattention, hyperactivity, and impulsivity [1,2]. Recent systematic reviews and meta-analyses estimate that ADHD affects approximately 8% of children and adolescents worldwide, although prevalence estimates vary according to age, diagnostic criteria, and methodological characteristics [3,4]. Attention difficulties, hyperactivity, and impulsivity associated with ADHD may adversely affect children’s academic performance, social functioning, emotional well-being, and quality of life [5,6]. It may also impair family functioning and impose a substantial psychological and economic burden through increased healthcare utilization, special education needs, social support requirements, and productivity losses [5,6]. Accordingly, the early identification and effective management of ADHD remain important public health priorities [5].

The core symptoms of ADHD may also be associated with altered eating behaviors that could influence habitual dietary intake [7,8]. Eating behaviors contribute to food selection, meal patterns, and the development of dietary habits during childhood, whereas diet quality and dietary patterns reflect broader characteristics of habitual intake [9,10]. Difficulties in executive functioning, reward processing, impulse control, and behavioral self-regulation have been proposed as potential mechanisms underlying differences in eating behaviors and food choices among children with ADHD [10,11,12,13]. Nevertheless, the relationships among ADHD, eating behaviors, and dietary characteristics remain incompletely understood, particularly regarding their temporal direction and underlying mechanisms [11,14].

Previous studies have linked ADHD diagnosis or elevated ADHD symptoms with greater food responsiveness, emotional overeating, and desire to drink, whereas findings concerning food fussiness have been less consistent [7,8,15,16]. However, some studies have identified differences in only selected eating behavior domains, whereas others have found no significant overall differences between children with ADHD and healthy controls [17,18].

Findings regarding dietary characteristics are similarly inconsistent. Western-like dietary patterns [19], processed food–sweets patterns [17] and sweet dietary patterns [20] have been positively associated with ADHD diagnosis or symptoms, whereas healthy dietary patterns have shown inverse associations [19,21]. More recently, greater consumption of ultra-processed foods has been prospectively associated with higher ADHD symptom scores, while greater consumption of unprocessed or minimally processed foods has shown an inverse association [22]. However, relatively few studies have concurrently evaluated multiple dimensions of eating behavior, diet quality, and dietary patterns in children with ADHD [23].

Furthermore, to the best of our knowledge, the study by Yan et al. (2023) is among the few studies to have evaluated dietary patterns and eating behaviors within the same sample and examined their respective associations with ADHD [17]. Consequently, associations between ADHD and multiple complementary indicators of eating behavior and diet, when evaluated within the same sample, remain insufficiently characterized [17,23].

Clinical heterogeneity represents another important limitation of the existing evidence [24]. Psychiatric comorbidity represents another important source of clinical heterogeneity in ADHD research. A recent systematic review and meta-analysis estimated that approximately 66.5% of children and adolescents with ADHD had at least one comorbid psychiatric disorder [24]. ADHD frequently co-occurs with autism spectrum disorder, anxiety, depression, tic disorders, and oppositional defiant disorder [24]. Because certain comorbid conditions are themselves associated with feeding problems and differences in dietary intake, their presence may complicate the attribution of observed dietary characteristics specifically to ADHD [25,26].

Psychostimulant treatment, particularly methylphenidate, may suppress appetite and contribute to weight loss in children and adolescents with ADHD [27].

Therefore, findings obtained from clinically heterogeneous or treated populations may reflect the effects of comorbidities and pharmacological treatment in addition to characteristics associated with ADHD. Studies involving newly diagnosed, treatment-naïve children without relevant comorbidities may help reduce these sources of heterogeneity.

Accordingly, this exploratory matched case–control study aimed to compare eating behaviors, diet quality, highly processed food consumption, and empirically derived dietary patterns between newly diagnosed, treatment-naïve children with ADHD who had no major neurodevelopmental, psychiatric, neurological, or chronic comorbidities and age- and sex-matched healthy controls.

## 2. Materials and Methods

### 2.1. Study Design

This observational, analytical, 1:1 pair-matched case–control study was conducted between February 2025 and February 2026 at the Child and Adolescent Psychiatry and Pediatrics outpatient clinics of Yalova University Training and Research Hospital, Yalova, Türkiye. Each child with ADHD was individually matched to a healthy control of the same sex and similar age, with a maximum difference of six months between their dates of birth. Matching was performed to minimize potential confounding by age and sex. The protocol was approved by the Ethics Committee for Non-Interventional Research in Health Sciences at Yalova Training and Research Hospital (Ethics number: 2024/397, Date 5 February 2025).

All procedures were carried out in accordance with the principles of the Declaration of Helsinki. Written informed consent was obtained from the parents or legal guardians of all participants.

### 2.2. Study Population

The case group comprised children aged 6–14 years who were newly diagnosed with ADHD at the Child and Adolescent Psychiatry Outpatient Clinic. ADHD diagnoses were established by child and adolescent psychiatrists according to the DSM-5 criteria following clinical interviews with the children and their parents or primary caregivers [28].

The clinical assessment included developmental and medical histories and evaluation of current psychiatric symptoms. The presence of additional neurodevelopmental, psychiatric, or neurological disorders was assessed and excluded by the child and adolescent psychiatrists during the diagnostic evaluation. Treatment-naïve status was defined as having received no previous pharmacological, psychosocial, or psychotherapeutic treatment for ADHD.

Children were eligible for the ADHD group if they: (1) were aged 6–14 years and met the DSM-5 diagnostic criteria for ADHD; (2) had no comorbid neurodevelopmental, psychiatric, or neurological disorder, including oppositional defiant disorder, conduct disorder, tic disorder, anxiety disorder, autism spectrum disorder, sleep disorder, Tourette syndrome, depression, or learning disability; (3) had no gastrointestinal malformation, inherited metabolic disorder, or chronic disease that could affect growth, nutritional status, dietary intake, or biochemical parameters; (4) were not using medications, vitamin or mineral supplements, or hormonal treatments and were not following a special diet; and (5) had no condition preventing anthropometric assessment; (6) did not require immediate symptomatic treatment before completion of the study assessments (clinical treatment was not postponed for study participation); (7) attended the clinic with a parent and both the child and parent were able to communicate in Turkish; and (8) provided written informed consent from the parent or legal guardian and assent from the child.

The control group comprised children aged 6–14 years attending the pediatrics outpatient clinic for routine health examinations during the same study period. Eligibility was evaluated by a pediatrician through parental report, review of available medical records, clinical interview, and physical examination. Children were eligible for the control group if they had no known neurodevelopmental, psychiatric, neurological, cognitive, metabolic, gastrointestinal, autoimmune, renal, hepatic, cardiovascular, or other chronic condition that could affect growth, nutritional status, dietary intake, or biochemical parameters. Children with an acute illness or infection potentially affecting biochemical measurements were excluded. Controls were also required not to use medications, dietary supplements, or hormonal treatments, not to follow a special diet, and to have no condition preventing anthropometric assessment. Controls who met the eligibility criteria and could be matched to a child with ADHD were selected from the pediatric outpatient clinic population. Each control was individually matched without replacement to one child with ADHD of the same sex and a date of birth within six months of that of the case.

### 2.3. Sample Size Estimation

The study had a multidimensional design and included multiple outcomes related to eating behaviors, dietary patterns, and diet quality. Therefore, one key outcome representing the main study hypotheses was selected as the reference variable for sample size estimation. The desire to drink subscale of the Children’s Eating Behaviour Questionnaire (CEBQ) was selected because it represented a clinically relevant eating behavior and comparable data were available from a previous study of children with ADHD. Based on the group means and standard deviations reported by Wu et al., the standardized mean difference was calculated as d = 0.538 [16]. An a priori power analysis was performed using G*Power version 3.1 (Heinrich Heine University Düsseldorf, Düsseldorf, Germany) for a two-sided comparison of two independent means, with a 1:1 allocation ratio, a type I error rate of 0.05, and 80% statistical power. The minimum required sample size was 112 participants, comprising 56 children with ADHD and 56 healthy controls. Because an estimate of the within-pair correlation was unavailable before data collection, the calculation was based on an independent-groups comparison. The final sample comprised 128 participants forming 64 age- and sex-matched pairs (Figure 1). Moreover, in the post hoc power analysis, based on the effect size obtained from the reference study (d = 0.538) [16], a two-tailed hypothesis, α = 0.05, and a 1:1 allocation ratio, the statistical power was calculated as 86.8% for a total sample of 128 participants, with 64 participants in each group.

### 2.4. Data Collection

During the recruitment period, all children newly diagnosed with ADHD at the Child and Adolescent Psychiatry Outpatient Clinic were consecutively screened for eligibility. The study was offered to every newly diagnosed, treatment-naïve child who met the eligibility criteria, and no eligible child was selectively approached by the researchers. Potential healthy controls were consecutively screened among children attending the Pediatrics Outpatient Clinic for routine health examinations or general health screening during the same period. Each child with ADHD was individually matched to one eligible control by sex and age. When more than one eligible control was available for a child with ADHD, the control with the closest age was selected. Recruitment continued until 64 eligible matched pairs had completed all study procedures. Eligible children and their parents or primary caregivers were referred to the principal researcher by a pediatrician or child and adolescent psychiatrist. The researcher provided detailed information about the study objectives, procedures, potential risks and benefits, the voluntary nature of participation and the right to withdraw from the study. Data collection began after written parental consent and age-appropriate child assent had been obtained.

Data were collected under comparable conditions and in the same sequence for both groups. First, venous blood samples were obtained by trained healthcare personnel at the hospital’s pediatric phlebotomy unit. Anthropometric measurements were then performed by the principal researcher using standardized procedures. Finally, standardized data collection instruments were administered to parents or primary caregivers by the principal researcher through face-to-face interviews. Participant recruitment, exclusion, matching, and inclusion in the final analysis are summarized in Figure 1.

### 2.5. Measurements and Assessment

#### 2.5.1. Demographics and Socioeconomic Assessment

Parents or primary caregivers provided information on the child’s date of birth and sex, parental marital status, and maternal education level. Parental marital status was categorized as married or divorced/widowed. Maternal education was classified as low (illiterate or primary school), medium (secondary or high school), or high (university or above).

Moreover, family material affluence was assessed using the four-item Family Affluence Scale-II (FAS-II), developed within the World Health Organization collaborative Health Behaviour in School-aged Children study and validated in Turkish [29,30]. The scale assesses family car ownership, whether the child has a separate bedroom, the frequency of family holidays, and the number of computers owned by the family. Item scores were summed to obtain a total score ranging from 0 to 9, with higher scores indicating greater family affluence. Total scores were categorized as low (0–2), medium (3–5), or high (6–9) family affluence. The Turkish version demonstrated a single-factor structure explaining 45.4% of the total variance and a composite reliability coefficient of 0.77 [30]. Parent-reported lifestyle characteristics included daily screen time, daily sleep duration, participation in regular physical activity (yes/no), and the frequency of unstructured outdoor play. Outdoor play frequency was categorized as low (never or 1–2 days/week), medium (3–4 days/week), or high (5–6 days/week or every day).

#### 2.5.2. Eating Behavioral Assessment

Children’s eating behaviors were assessed using two complementary parent-reported instruments: Children’s Eating Behaviour Questionnaire (CEBQ) and Behavioral Pediatrics Feeding Assessment Scale (BPFAS) [31,32]. The CEBQ was used to assess appetitive traits and habitual eating tendencies, whereas the BPFAS was used to evaluate parent-reported mealtime and feeding behaviors. Both instruments were administered to parents or primary caregivers through face-to-face interviews.

*Children’s Eating Behavior Questionnaire (CEBQ):* The CEBQ is a parent-reported instrument developed to assess eating behaviors in children and consists of 35 items rated on a five-point Likert scale ranging from 1 (never) to 5 (always) [31]. The Turkish version of the CEBQ was validated by Yılmaz et al., and it was reported that the eight-factor structure explained 58.2% of the total variance, with Cronbach’s alpha coefficients ranging from 0.61 to 0.84 [33]. The CEBQ assesses eight dimensions of eating behavior that can be grouped into two broader domains: food-avoidant eating behaviors (satiety responsiveness, slowness in eating, food fussiness, and emotional undereating) and food-approach eating behaviors (food responsiveness, enjoyment of food, desire to drink, and emotional overeating). Subscale scores were calculated as the mean of the corresponding item scores, resulting in scores ranging from 1 to 5. Higher scores indicated a stronger expression of the respective eating behavior.

*Behavioral Pediatrics Feeding Assessment Scale (BPFAS):* The BPFAS is a parent-reported instrument developed by Crist et al. to assess problematic feeding behaviors in children [32]. The Turkish adaptation and validation study conducted by Onal et al. demonstrated good psychometric properties, with a Cronbach’s alpha coefficient of 0.88 and test–retest reliability of 0.88 [34]. The Turkish version consists of 24 items grouped into four dimensions: food selectivity (7 items), early food refusal (5 items), early texture-related food refusal (5 items), and late food refusal (7 items). All items are rated on a five-point Likert scale ranging from 1 (never) to 5 (always). Subscale scores were calculated by summing the corresponding item scores after reverse-scoring the positively worded items. Higher scores indicated a greater frequency of problematic feeding behaviors.

#### 2.5.3. Dietary Assessment

Habitual dietary intake, diet quality, ultra-processed food consumption, and dietary patterns were evaluated using complementary assessment approaches. Diet quality was assessed using the MIND (Mediterranean–DASH Intervention for Neurodegenerative Delay) Diet Index [35], ultra-processed food consumption was evaluated using the Screening Questionnaire of Highly Processed Food Consumption (sQ-HPF) [36]. In addition, dietary patterns were derived using principal component analysis (PCA) to identify food groups commonly consumed together and thereby characterize overall dietary behavior beyond the consumption of individual foods.

Food and beverage intake was assessed using semi-quantitative food frequency and beverage frequency questionnaires, for which validity and reliability had previously been evaluated [37]. The questionnaires were administered by the researcher through face-to-face interviews with the children and their parents or primary caregivers and were used to assess the children’s habitual food and beverage consumption over the preceding month. Information regarding foods consumed at home was obtained primarily from the parents or primary caregivers, whereas children provided complementary information on foods and beverages consumed outside the home, particularly at school. The food frequency questionnaire included six response categories: ≥2 times/day, once/day, 3–6 times/week, 1–2 times/week, 1–3 times/month, and less than once/month or never. The beverage frequency questionnaire also included six response categories: ≥5 times/day, 3–4 times/day, 1–2 times/day, 3–6 times/week, 1–2 times/week, and rarely or never [37]. Reported consumption frequencies were converted into daily equivalents and multiplied by the reported portion sizes to estimate habitual daily intake in grams or milliliters. Standard portion sizes were determined according to the Turkish Ministry of Health Nutrition Guidelines [38], and photographs from the Food and Meal Photograph Catalogue were used to facilitate portion-size estimation [39]. Daily energy and nutrient intakes were calculated using the Nutrition Information System software (BeBIS version 8.1; Blue Apple Software, Istanbul, Türkiye). Extreme observations were checked against the original food-frequency responses and reported portion sizes, and no participants were excluded because of implausible energy intake.

*MIND Diet Index*: The MIND diet is a hybrid dietary pattern combining components of the Mediterranean and DASH diets and was originally developed to support brain health and reduce the risk of neurodegenerative diseases [40]. In the present study, adherence to the MIND diet was assessed using the scoring method applied to children by Bayranj et al. (2025) [35], which was based on the original MIND Diet Index developed by Morris et al. (2015) [40]. The index included nine brain-healthy food groups—green leafy vegetables, other vegetables, nuts, berries, legumes, whole grains, fish, poultry, and olive oil—and five food groups considered less favorable for brain health—red meat, cheese, butter or margarine, pastries and sweets, and fast or fried foods. The wine component of the original index was excluded because it was not applicable to the pediatric study population. Participants were categorized into tertiles of consumption for each component based on the distribution in the overall study sample. Brain-healthy components were assigned scores of 1, 2, and 3 from the lowest to the highest tertile, respectively, whereas less favorable components were reverse-scored. Component scores were summed to obtain a total MIND Diet Index score ranging theoretically from 14 to 42, with higher scores indicating greater adherence according to the sample-based MIND Diet Index.

*The Screening Questionnaire of Highly Processed Food Consumption (sQ-HPF):* Highly processed food consumption was assessed using the Turkish version of the sQ-HPF, with permission from the developers of the Turkish adaptation. The sQ-HPF was originally developed by Martinez-Perez et al. as a brief screening instrument integrating criteria from four food-processing classification systems [41]. The Turkish version comprises 11 commonly consumed highly processed food and beverage groups and demonstrated moderate internal consistency (Cronbach’s α = 0.65) and good test–retest reliability (ICC = 0.76) in its adult validation sample [36]. Moreover, before data collection, the comprehensibility and feasibility of the sQ-HPF for use in children were pilot-tested in a separate group of 33 healthy children in the target age range attending the pediatric outpatient clinic at the same hospital. In the present study, the questionnaire was administered face-to-face by the researcher to the child and parent or primary caregiver together. Parents or caregivers provided information on foods consumed at home, whereas children provided complementary information regarding foods and beverages consumed outside the home, particularly at school. When necessary, the researcher provided standardized explanations and age-appropriate examples of the food categories without changing the original scoring criteria. Each item was scored as 0 or 1 according to whether the reported consumption frequency met the predefined threshold, resulting in a total score ranging from 0 to 11, with higher scores indicating a greater tendency toward highly processed food consumption [36].

*Dietary pattern assessment:* Dietary patterns were derived using principal component analysis (PCA). The 55 food items and 16 beverage items included in the FFQ and BFQ were grouped into nine food groups according to their nutritional characteristics and degree of processing, based on previous studies (Table S1) [17,19]. To minimize the influence of differences in total energy intake, food-group intakes were energy-adjusted using the density method, expressed as grams or milliliters per 1000 kcal, and subsequently standardized as z-scores. The suitability of the data for PCA was supported by a Kaiser–Meyer–Olkin value of 0.702 and a statistically significant Bartlett’s test of sphericity (χ^2^ = 135.158, df = 36, *p* < 0.001). Varimax rotation was applied to improve the interpretability of the retained components. The number of components retained was determined by considering eigenvalues greater than 1.0, visual inspection of the scree plot, and parallel analysis. Food groups with absolute factor loadings ≥0.30 were considered to contribute meaningfully to the corresponding dietary pattern. Factor scores were calculated using the regression method and used as continuous indicators of individual adherence to each dietary pattern in subsequent analyses.

#### 2.5.4. Anthropometric Measurement and Assessment

All anthropometric measurements were performed by the principal researcher according to standardized procedures [42]. Height was measured to the nearest 0.1 cm using a Leicester portable stadiometer (seca 213; seca GmbH & Co. KG, Hamburg, Germany), with participants standing barefoot on a flat surface, feet together, body upright, and head positioned in the Frankfurt horizontal plane [42]. Waist circumference was measured midway between the lowest rib and the iliac crest, hip circumference at the widest point over the buttocks, and mid-upper arm circumference at the midpoint between the acromion and olecranon processes [42]. Circumference measurements were obtained twice using a non-elastic measuring tape (Medikaltec6310; Medikaltec, Kayseri, Türkiye), and the mean of the two measurements was used in the analyses. Body weight and body fat percentage were measured using a bioelectrical impedance analyzer (Tanita InnerScan BC-532; Tanita Corp., Tokyo, Japan). Measurements were performed with participants wearing light clothing and standing barefoot on the device after removing their shoes, socks, and metallic accessories. Age, sex, and height were entered into the device before measurement. Body mass index (BMI) was calculated as body weight in kilograms divided by height in meters squared (kg/m^2^) [43]. BMI-for-age z-scores (BAZ) were calculated using WHO AnthroPlus software version 1.0.4 (World Health Organization, Geneva, Switzerland) and evaluated according to the WHO Growth Reference for school-aged children and adolescents aged 5–19 years [43].

#### 2.5.5. Blood Sampling and Biochemical Analyses

Approximately 10 mL of venous blood was collected from each participant in the morning after an overnight fast at the hospital’s pediatric phlebotomy unit by trained healthcare personnel following standard venipuncture procedures. Blood samples were processed and analyzed in the hospital laboratory according to routine clinical protocols.

#### 2.5.6. Covariates

Age and sex are important sources of variation in ADHD prevalence and diagnostic recognition [4,44]. These variables were controlled at the study-design stage through 1:1 pair matching, with a maximum age difference of six months within each matched pair.

The matched structure was subsequently accounted for in the association analyses using conditional logistic regression. Covariates were selected primarily based on their conceptual relevance, temporal relationship to the exposure and outcome assessments, and susceptibility to reverse causality. Between-group differences were also considered, but covariate selection was not based solely on statistical significance.

Maternal education, a relatively stable indicator of family socioeconomic position, has been associated with children’s healthy eating practices and dietary patterns [45,46]. Evidence from multinational birth cohorts, population-based register studies, and family-based analyses has shown associations between lower maternal education and childhood ADHD [47,48,49]. Maternal education has additionally been reported to potentially modify the relationship between children’s dietary patterns and ADHD symptoms [50]. Maternal education generally precedes the child’s current ADHD and dietary assessments, is relatively stable, and was considered less susceptible to reverse causality than the other measured covariates. It was therefore included as the sole covariate in the parsimonious primary adjusted model because of its potential role as a confounder (Model 2).

Current family affluence was considered a potential source of confounding, as previous studies suggest that socioeconomic circumstances may be related to both children’s dietary patterns [46] and ADHD status or risk [51,52,53]. However, the magnitude, duration, and timing of socioeconomic disadvantage may be relevant to its relationship with ADHD [52]. In the present study, FAS-II reflects current family affluence and does not capture the timing or persistence of socioeconomic conditions.

Parental marital status may reflect multiple family-related processes and may also be interrelated with family affluence. Because its association with ADHD may be bidirectional, including evidence that childhood ADHD may precede subsequent family separation [54], its temporal position was considered less clear than that of maternal education.

Given their potential confounding roles and the observed between-group differences, FAS-II and parental marital status were included alongside maternal education in the expanded sensitivity model (Model 3).

Although unstructured outdoor play differed between the groups, it was not included as a covariate because it may reflect ADHD-related behavior rather than a pre-existing confounder, and its temporal relationship with ADHD was uncertain. Given the limited number of matched pairs, this staged modeling strategy was used to balance confounding control and model parsimony.

#### 2.5.7. Statistical Analysis

All statistical analyses were performed using IBM SPSS Statistics for Windows, version 22.0 (IBM Corp., Armonk, NY, USA). The distributions of continuous variables and within-pair differences were evaluated using histograms, normal probability plots, and the Shapiro–Wilk test. Continuous variables were summarized as mean ± standard deviation or median (Q1–Q3), as appropriate, whereas categorical variables were presented as numbers and percentages, n (%). Continuous variables were compared using the Wilcoxon signed-rank test. Binary categorical variables were compared using McNemar’s exact test, whereas categorical variables with more than two levels were evaluated using the McNemar–Bowker symmetry test.

For the group-comparison analyses, each set of related outcomes derived from the same questionnaire or analytical domain including questionnaire subscales, post hoc item-level comparisons within a subscale, and data-derived dietary patterns was treated as a separate family of comparisons. Multiplicity correction was applied within each family to control the family-wise Type I error rate. Accordingly, Bonferroni correction was applied separately across the eight CEBQ subscale comparisons and the four BPFAS subscale comparisons. It was also applied across the seven post hoc, exploratory item-level comparisons conducted within the late food refusal subscale solely for hypothesis-generating purposes. Holm correction was applied across the three dietary patterns derived from the same PCA. As the MIND Diet Index and sQ-HPF each yielded a single summary score representing a distinct dietary construct, no multiplicity correction was applied to these comparisons.

For the regression analyses, a broader multiplicity approach was used because all exposure measures were evaluated in parallel in relation to the same outcome, ADHD status, and contributed to the principal inferential findings. Thus, within each model specification, the six continuously scored exposure measures—CEBQ desire to drink, MIND Diet Index, sQ-HPF, and the three PCA-derived dietary patterns—were treated as one family of regression tests. Thus, within each model specification, the six continuously scored exposure measures—CEBQ desire to drink, MIND Diet Index, sQ-HPF, and the three PCA-derived dietary patterns—were treated as one family of regression tests.

The six corresponding median-dichotomized analyses were treated as a separate family of alternative categorical parameterizations. Within each model, Holm correction was applied separately to the six continuous and six median-dichotomized analyses. Both nominal and Holm-adjusted *p*-values are reported, with statistical inference based on the adjusted values.

Given the 1:1 age- and sex-matched case–control design, conditional logistic regression was used to account for the dependence within matched pairs. Because the analyses were conducted in SPSS, which does not provide a dedicated conditional logistic regression procedure, the analysis was implemented using the mathematically equivalent stratified Cox regression formulation. A constant artificial time was assigned to all participants, ADHD status was specified as the event indicator, and the matched-pair identifier was entered as the stratification variable. With one case per matched pair, the Cox partial likelihood is equivalent to the conditional logistic likelihood; therefore, the exponentiated coefficients represent matched odds ratios.

Each eating-behavior or dietary exposure was entered separately into the conditional logistic regression models. Thus, the resulting estimates represent exposure-specific associations with ADHD rather than mutually adjusted independent associations. Continuous score analyses constituted the primary analyses. The same six exposure measures were additionally dichotomized at their sample medians and evaluated as secondary analyses, with values at or below the median used as the reference category. Matched odds ratios and their 95% confidence intervals were reported. Model 1 was the unadjusted matched model. Model 2, designated as the primary adjusted model, included maternal education as the sole covariate. This parsimonious specification was selected based on the temporal considerations described above, the limited number of matched pairs, and the need to avoid model overparameterization. Model 3 was an expanded sensitivity model that included maternal education, the FAS-II total score, and parental marital status to evaluate the robustness of the estimates to further adjustment for potential confounding.

## 3. Results

### 3.1. Characteristics of Participants

The characteristics of the participants are presented in Table 1. The ADHD and control groups were matched by age and sex. The proportion of children whose parents were divorced or widowed was higher in the ADHD group than in the control group. Although the median FAS-II total score did not differ between the groups, the distribution of family-affluence categories differed significantly, with a higher proportion of children in the ADHD group classified in the high-affluence category.

Children with ADHD also had a higher frequency of unstructured outdoor play. Ferritin concentrations were significantly lower, whereas total iron-binding capacity was significantly higher, in children with ADHD; the remaining biochemical parameters did not differ significantly between the groups.

Moreover, among the anthropometric measures, mid-upper arm circumference was higher in the ADHD group than in the control group (median [Q1–Q3]: 19.0 [17.0–21.8] vs. 18.0 [17.0–22.0] cm; *p* = 0.015), whereas height, weight, BMI-for-age z-score, waist-to-height ratio, and body fat percentage did not differ significantly between the groups.

### 3.2. Comparison of Eating Behaviors, Diet Quality and Dietary Patterns

#### 3.2.1. Eating Behavior Characteristics

Multiplicity correction was applied to control the family-wise Type I error rate for these multiple subscale comparisons. Bonferroni correction was applied separately to the subscales of each questionnaire, with adjusted significance thresholds of *p* < 0.00625 for the CEBQ and *p* < 0.0125 for the BPFAS. After correction, only the CEBQ desire to drink subscale and the BPFAS late food refusal subscale remained significantly higher in children with ADHD than in matched controls (both *p* < 0.001). No significant between-group differences were observed in the remaining CEBQ or BPFAS subscales (Table 2).

Moreover, post hoc item-level comparisons were conducted for exploratory and hypothesis-generating purposes because the individual items of the BPFAS late food refusal subscale reflect distinct mealtime behaviors. Before correction, children with ADHD had nominally higher scores for delays eating by talking (*p* = 0.020), prefers liquids instead of solid foods (*p* < 0.001), and argues about what he/she will or will not eat (*p* = 0.012) than matched controls. After Bonferroni correction for seven comparisons (adjusted significance threshold, *p* < 0.0071), only prefers liquids instead of solid foods remained statistically significant (Table S2). These item-level findings should be interpreted as hypothesis-generating.

#### 3.2.2. Dietary Characteristics

The distributions of the MIND Diet Index and sQ-HPF scores are presented in Figure 2. Children with ADHD had lower MIND Diet Index scores than matched controls (median: 25.0 vs. 27.0, respectively; *p* < 0.001), whereas their sQ-HPF scores were higher (median: 6.5 vs. 4.0, respectively; *p* < 0.001).

According to the BFQ, children with ADHD also reported higher daily water intake than controls (median: 1500 vs. 1000 mL/day; *p* < 0.001). Energy drink and ayran/kefir consumption were nominally higher in the ADHD group (*p* = 0.039 and *p* = 0.011, respectively), whereas carbonated soft drink consumption did not differ significantly between the groups (*p* = 0.087).

#### 3.2.3. Dietary Patterns

The PCA identified three dietary patterns, which together explained 53.9% of the total variance. The first, second, and third components explained 21.1%, 17.8%, and 15.0% of the variance, respectively. The three-component solution was also supported by visual inspection of the scree plot and parallel analysis. The patterns were descriptively labeled “Healthy dietary pattern,” “Processed foods and sugar-sweetened beverages dietary pattern,” and “Grain- and fruit-based dietary pattern” according to the food groups with the highest factor loadings (Table S3). The Healthy dietary pattern score was significantly lower in the ADHD group than in the control group (median [IQR]: −0.36 [1.39] vs. 0.12 [1.38]; unadjusted *p* = 0.009). This difference remained statistically significant after Holm correction for the three dietary-pattern comparisons (adjusted *p* = 0.027). No significant between-group differences were observed for the processed foods and sugar-sweetened beverages dietary pattern (−0.34 [1.38] vs. −0.16 [1.16]; unadjusted *p* = 0.968; Holm-adjusted *p* = 1.000) and the Grain- and fruit-based dietary pattern (−0.24 [1.42] vs. −0.11 [0.97]; unadjusted *p* = 0.602; Holm-adjusted *p* = 1.000).

#### 3.2.4. Associations of Eating Behaviors, Diet Quality and Dietary Patterns with ADHD

The associations of eating behaviors, dietary indices, highly processed food consumption, and dietary patterns with ADHD status across the three models are presented in Table 3.

In the unadjusted model (Model 1), higher desire to drink and sQ-HPF scores were associated with higher odds of ADHD, whereas a higher MIND Diet Index score was associated with lower odds; these associations remained significant after Holm correction in both continuous and median-dichotomized analyses (Holm-adjusted *p* ≤ 0.020). The nominal inverse association observed for the continuous healthy dietary pattern did not remain significant after Holm correction (nominal *p* = 0.027; Holm-adjusted *p* = 0.081). No statistically significant associations were observed for the other dietary patterns.

In the parsimonious primary adjusted model (Model 2), higher desire-to-drink scores (OR = 1.88, 95% CI: 1.37–2.58) and sQ-HPF scores (OR = 2.44, 95% CI: 1.61–3.71) were associated with higher odds of ADHD, whereas higher MIND Diet Index scores were associated with lower odds (OR = 0.77, 95% CI: 0.66–0.89). These associations, observed in separate exposure-specific models adjusted for maternal education, remained statistically significant after Holm correction (adjusted *p* ≤ 0.003; Table 3 and Figure 3).

Model 3 was conducted as an expanded sensitivity analysis to examine the robustness of the results to additional adjustment for the FAS-II total score and parental marital status. The associations of desire to drink, MIND Diet Index, and sQ-HPF with ADHD remained directionally consistent and statistically significant after Holm correction. The continuous healthy dietary pattern was also inversely associated with ADHD in this sensitivity model (OR = 0.54, 95% CI: 0.33–0.88; Holm-adjusted *p* = 0.041), whereas the corresponding median-dichotomized analysis was not statistically significant after Holm correction (adjusted *p* = 0.091).

## 4. Discussion

To the best of our knowledge, this exploratory matched case–control study evaluated eating behaviors, diet quality, highly processed food consumption, and dietary patterns in children with newly diagnosed, treatment-naïve ADHD without neurodevelopmental, psychiatric, neurological, or chronic comorbidities and in age- and sex-matched healthy controls. Overall, children with ADHD showed a greater desire to drink and a less favorable dietary profile, characterized by lower sample-based MIND Diet Index scores and higher sQ-HPF scores, than matched controls.

### 4.1. Participant Characteristics, Anthropometric and Biochemical Findings

In this study, in addition to the eating behavior and dietary analyses, several sociodemographic and lifestyle characteristics, anthropometric measurements, and some nutrition-related biochemical parameters were examined descriptively to provide a broader clinical context.

Previous studies suggest that psychosocial and family-context factors, including socioeconomic disadvantage, parental separation, and single-parent family structure, may be related to childhood ADHD symptoms and diagnosis [51,52,53]. In the present study, having a divorced or widowed parent was more frequent in the ADHD group than in the control group, broadly aligning with this literature [51,53]. However, this difference was based on a small number of participants (eight children in the ADHD group and one control) and was not a primary study outcome; it should therefore be regarded as a descriptive observation that neither establishes temporal direction nor supports a causal interpretation.

On the other hand, the categorical classification of the FAS-II showed a higher proportion of children in the high-affluence category in the ADHD group. This descriptive finding differs from the general pattern in the literature suggesting that socioeconomic disadvantage may be related to childhood ADHD [51,52].

This finding can be explained within this context. The study was conducted in a relatively small, non-metropolitan city. The study hospital is the city’s only teaching and research hospital and its principal referral center, with the largest number of specialists in the relevant clinical fields. Although the hospital serves families from different socioeconomic backgrounds, families with greater socioeconomic resources, including better transportation opportunities, may have had easier access to specialist assessment in a relatively small city. These factors may have influenced which families reached specialist services and were subsequently included in the clinical sample. Thus, the higher proportion of children from high-affluence families in the ADHD group may reflect a greater likelihood of these families reaching specialist psychiatric services and subsequently being included in the clinical sample, rather than a higher occurrence of ADHD among children with greater family affluence.

In the present study, the groups did not differ in regular physical activity, whereas frequent unstructured outdoor play was more common in the ADHD group. Findings regarding physical activity among children with ADHD have varied across studies, possibly reflecting differences between planned or organized activities and unstructured or free-living movement [55,56,57,58]. Some studies have reported lower participation in organized activities [55,56,57], whereas accelerometer-based research has indicated higher free-living activity, particularly among children with hyperactive/impulsive or combined ADHD presentations [58]. This contrast may reflect the challenges that difficulties involving attention, impulse control, motor skills, and self-regulation can pose for organized participation, while greater spontaneous movement may be related to motor hyperactivity [57,58]. However, this evidence remains limited and does not establish a causal explanation for the present finding. Because physical activity and outdoor play were parent-reported, were not objectively quantified, and were not primary study outcomes, these results should be regarded as descriptive and hypothesis-generating and require confirmation using objective activity measurements.

In the present study, selected nutrition-related biochemical markers were also examined descriptively. Among these markers, children with ADHD had lower ferritin concentrations and higher total iron-binding capacity than matched controls, whereas no significant between-group difference was observed in serum iron. The roles of iron in neurodevelopment, myelination, and catecholamine synthesis have provided a biological rationale for investigating iron status in children with ADHD, and this relationship has been examined in previous studies [59,60]. A meta-analysis similarly reported lower serum ferritin concentrations in children with ADHD but no significant differences in serum iron or transferrin [60]; however, a subsequent systematic review found that results for peripheral ferritin and serum iron were inconsistent across studies [59]. The pattern observed in the present study may be compatible with lower iron stores but does not establish iron deficiency or an ADHD-related biological mechanism. Peripheral iron markers may be influenced by the dietary sources and bioavailability of iron, intestinal absorption, metabolic utilization, growth-related requirements, inflammation, and other physiological factors. In view of their descriptive, non-primary nature and the cross-sectional study design, these biochemical findings warrant cautious interpretation.

Overall, the groups had comparable anthropometric profiles, with no significant between-group differences in height, weight, BMI-for-age z-score, waist-to-height ratio and body fat percentage. The observed similarity in height, weight, and BMI is consistent with findings from several case–control studies that reported no significant differences in these measures between children with ADHD and healthy controls [17,61]. In contrast, other studies have reported greater body fat mass among children with ADHD and differences between ADHD and control groups in the prevalence of malnutrition, overweight, and obesity [16,62]. These differences across studies may be related to variations in age, sex distribution, medication use, comorbidities, matching procedures, and the anthropometric definitions applied. In the present study, mid-upper arm circumference was the only anthropometric measure that was higher in the ADHD group. However, because mid-upper arm circumference reflects both muscle and subcutaneous fat and this finding was not accompanied by differences in BMI-for-age z-score, waist-to-height ratio, or body fat percentage, it should not be interpreted as evidence of greater adiposity or a distinct nutritional status. Furthermore, individual matching by age and sex, which resulted in a mean age difference of approximately one month between the groups, enabled the children to be compared at similar stages of growth and development and may have reduced age- and sex-related variability in the anthropometric measures.

### 4.2. Eating Behaviors and ADHD

The principal eating-behavior finding was the higher desire-to-drink score among children with ADHD, with similar associations observed across the parsimonious adjusted and expanded sensitivity models. Moreover, higher late-food-refusal scores were observed as a secondary finding. As a hypothesis-generating observation, the greater tendency to prefer liquids over solid foods was consistent with a beverage-oriented eating tendency among children with ADHD.

Previous case–control studies using the CEBQ have reported heterogeneous findings regarding eating behaviors among children with ADHD [15,16,17,18,63]. Consistent with the present findings, a Turkish study comparing newly diagnosed, treatment-naïve children with ADHD with healthy controls of similar age and sex found that desire to drink was the only CEBQ subscale that differed significantly between the groups [63]. In another Turkish study, in which psychiatric comorbidities were not specified as an exclusion criterion and approximately 18% of children with ADHD were receiving medication, higher scores were reported for food responsiveness, emotional overeating, desire to drink, and food fussiness [15]. A study conducted in China similarly reported higher scores for desire to drink and several other food-approach dimensions among children with ADHD, although medication use and psychiatric comorbidities were not clearly reported [16]. In contrast, studies conducted in Iran and China found no significant differences in direct between-group comparisons of CEBQ subscale scores [17,18]. Nevertheless, in the Chinese study, higher desire-to-drink scores, when dichotomized at the sample median, were associated with higher odds of ADHD after adjustment for potential confounders [17].

Taken together, these varying findings may partly reflect differences in the clinical characteristics of the study populations. The present findings add to the emerging evidence of an association between desire to drink and ADHD in a carefully characterized clinical sample, although confirmation in larger independent samples is needed.

Evidence based on the BPFAS is considerably more limited. In a comparative cross-sectional study conducted in Malaysia, Sha’ari et al. reported that leaving the table during meals, taking longer than 20 min to complete a meal, being reluctant to try new foods, and having tantrums during meals were more common among children with ADHD than among typically developing children [64]. The occurrence of different BPFAS items across studies may reflect variation in the mealtime behaviors reported among children with ADHD. Moreover, given their exploratory and hypothesis-generating nature, these item-level findings require confirmation in larger independent samples.

The inconsistencies across studies may partly reflect differences in participants’ age and clinical characteristics, the presence of psychiatric or neurodevelopmental comorbidities, treatment status, and the methods used to assess eating behaviors. Co-occurring neurodevelopmental and psychiatric conditions may also be related to eating behaviors and dietary intake [25,65]. For example, sensory sensitivities, food selectivity, and food refusal have been reported more frequently in children with autism spectrum disorder, which may co-occur with ADHD [25,65]. In addition, psychostimulant treatment, particularly methylphenidate, has been associated with decreased appetite and weight loss in children and adolescents with ADHD [27]. By including newly diagnosed, treatment-naïve children without relevant comorbidities, the present study may have reduced some of these sources of clinical heterogeneity, although other measured and unmeasured influences cannot be excluded.

In the present study, the higher desire-to-drink scores observed among children with ADHD may be interpreted in the context of several non-mutually exclusive and potentially bidirectional processes involving interactions between child-level developmental and behavioral characteristics and familial and environmental factors. In this context, executive-function and self-regulatory difficulties have been proposed as possible correlates of eating-behavior dysregulation in ADHD and in pediatric populations more broadly [11,66]. Similarly, sensory and oral-processing differences reported among children with ADHD may provide a broader context for interpreting the beverage-related findings, although their specific relevance to desire to drink remains uncertain [67]. Within this framework, beverages, which generally require less oral processing than solid foods and tend to be easier to consume and more homogeneous in texture, may be more readily accepted by some children who have difficulty sustaining attention and behavioral engagement during meals. However, these explanations should be regarded as conceptual possibilities rather than mechanisms supported by our findings because executive functioning, self-regulation, and sensory processing were not directly assessed in the present study.

In addition, eating behavior during childhood is a complex and multifactorial construct shaped by interactions among biological, developmental, psychological, familial, and environmental factors [68]. In this context, factors not assessed in the present study, including parental dietary habits and mental health, children’s prior food experiences, family mealtime routines, parental role modeling and feeding practices, eating in front of screens, the availability of foods and beverages at home, and socioeconomic and cultural conditions, may partly account for the observed association. Although the analyses accounted for the FAS-II total score and maternal education, these variables may not fully capture the broader familial and environmental context; therefore, residual confounding from unmeasured factors cannot be excluded. Moreover, reciprocal relationships between children’s eating behaviors and parental feeding practices over time have also been reported [69]. Within this framework, parents may respond to the mealtime behaviors of children with ADHD by offering beverages that are more readily accepted, while such adaptations may, in turn, contribute to the maintenance of the child’s existing preferences.

Given the observational design of the study, reverse causality cannot be excluded. Although the sample comprised newly diagnosed, treatment-naïve children, this does not imply that ADHD symptoms had only recently emerged. ADHD-related neurodevelopmental and contextual characteristics may therefore have shaped children’s eating behaviors over time.

It is important to emphasize that the observational design does not allow the temporal direction or causality of the observed association to be determined. Accordingly, the proposed explanations should be regarded as conceptual frameworks for interpreting the observed association rather than as established mechanisms. The direction of the association and its potential underlying processes require further examination in larger independent samples, particularly in prospective studies.

### 4.3. Diet Quality, Highly Processed Food Consumption and Dietary Patterns and ADHD

Regarding dietary characteristics, children with ADHD had lower MIND Diet Index and Healthy dietary pattern scores and higher sQ-HPF scores than matched controls. In the exposure-specific models, the associations observed for the MIND Diet Index and sQ-HPF remained statistically significant after adjustment and Holm correction (adjusted model and expanded sensitivity model). However, the inverse association observed for the Healthy dietary pattern did not remain statistically significant after Holm correction in the parsimonious adjusted model. Therefore, this finding should be considered exploratory and interpreted cautiously.

In the present study, the MIND Diet Index was used as a measure of diet quality potentially relevant to brain health in a neurodevelopmental context. To our knowledge, evidence regarding the MIND diet and childhood ADHD remains limited. In an Iranian case–control study, Bayranj et al. reported that children in the highest quartile of a sample-based MIND Diet Index had lower odds of ADHD than those in the lowest quartile [35]. Even though this finding is similar in direction to the lower MIND Diet Index scores observed among children with ADHD in the present study, the use of sample-derived cut-off points limits the external interpretability of the absolute scores and direct comparisons between studies.

Moreover, highly processed food consumption was assessed using the sQ-HPF, which captures consumption frequency across predefined categories of highly processed foods and beverages. In the present sample, higher continuous sQ-HPF scores were observed among children with ADHD than among matched controls. In the exposure-specific models, the corresponding positive association with ADHD status remained statistically significant after adjustment and Holm correction; however, this finding should be interpreted cautiously in light of the measurement-related limitations of the sQ-HPF in this pediatric population. Directly comparable evidence assessing highly processed food consumption in childhood ADHD using measures specifically designed for this purpose remains quite limited. Although differing from the present study in terms of study design, dietary assessment method, and ADHD ascertainment, a nationally representative cross-sectional study of Israeli children reported higher NOVA-classified UPF consumption among children with a parent-reported physician diagnosis of ADHD [70]. Similarly, in a population-based Brazilian birth cohort, repeatedly assessed higher UPF consumption was associated with higher hyperactivity/inattention symptom scores [22]. In contrast, a small cross-sectional study of Turkish children without clinically diagnosed ADHD found no significant association between NOVA-based UPF consumption and parent-reported ADHD symptom scores [71]. Taken together, the limited available evidence is not entirely consistent. Differences in study design, ADHD ascertainment, dietary assessment methods, sample characteristics, and sample size may partly account for the varying findings and limit direct comparisons across studies; moreover, the observational nature of the evidence precludes causal interpretation.

In the present study, dietary patterns were also explored using PCA. Dietary patterns derived using PCA are data-driven, sample-specific constructs that identify foods or food groups that tend to be consumed together within a given study population, rather than measuring adherence to a predefined dietary model [72]. Accordingly, across studies examining dietary patterns in relation to ADHD among children and adolescents, the number, composition, labeling, and total variance explained by the derived patterns may differ depending on the dietary assessment method, variables entered into the analysis, food-grouping strategy, analytical decisions, and population-specific dietary characteristics [14,17,21,72,73].

Previous case–control studies conducted in China, Iran, South Korea, and Spain have generally reported lower odds of ADHD or lower ADHD prevalence in relation to greater adherence to healthy, traditional–healthy, fish–white-meat, or vegetable-oriented dietary patterns [19,21,73,74]. Prospective evidence from South Korea similarly indicated that greater adherence to a vegetable dietary pattern at four years of age was associated with fewer parent-reported ADHD symptoms at six years [20]. These findings are broadly similar in direction to the lower Healthy dietary pattern scores observed among children with ADHD in the present study. However, because the corresponding inverse association did not remain statistically significant after Holm correction in the parsimonious adjusted model, our finding should be regarded as exploratory. Findings for Western, processed-food, snack, and sweet dietary patterns have been less consistent. Positive associations with ADHD or ADHD symptoms have been reported for processed food–sweets, Western, sweet, or fast-food patterns in several studies [17,20,74,75]. In contrast, no significant or consistent associations were observed for the fast food–sweet pattern reported by Zhou et al., the snack pattern reported by Woo et al., or the Sweet pattern reported by Rojo- Marticella et al. [19,21,74].

Differences across studies may be related to participants’ clinical characteristics, culturally specific dietary practices, dietary assessment tools, and analytical approaches. It should also be recognized that PCA-derived patterns are sensitive to the selection and grouping of input variables, that broad food groups may combine nutritionally heterogeneous components, and that the resulting patterns are data- and population-specific. These features limit both the interpretation of the identified patterns and direct comparisons across studies. Given that the inverse association observed for the Healthy dietary pattern in the present study did not remain statistically significant after Holm correction, this finding should be regarded as exploratory and evaluated in larger, independent, and prospective studies.

Moreover, the differing findings for the sQ-HPF and the PCA-derived Processed foods and sugar-sweetened beverages pattern may reflect the distinct dietary dimensions captured by the two approaches. The sQ-HPF assesses consumption frequency across predefined categories of highly processed foods and beverages, whereas the PCA-derived pattern represents a sample-specific combination of food groups consumed together and also incorporates contributions from other food groups. Accordingly, the pattern score does not represent an estimate of overall highly processed food consumption, which may partly explain why the findings obtained using the two approaches did not coincide.

When considered together, the findings for the MIND Diet Index and sQ-HPF suggest that less favorable characteristics may have been observed in specific dimensions of diet among children with ADHD. However, the absence of an association between ADHD and the Processed foods and sugar-sweetened beverages pattern, together with the failure of the inverse association observed for the Healthy dietary pattern to remain statistically significant after Holm correction, limits the interpretation of the findings as reflecting a generalized “unhealthy dietary pattern.” These exploratory findings should therefore be interpreted within the specific and distinct dietary dimensions captured by the respective assessment approaches.

How these dietary dimensions may be related to ADHD remains uncertain. More frequent consumption of highly processed foods may displace nutrient-dense foods from the diet and coincide with higher intakes of refined carbohydrates, saturated fat, sodium, and certain food additives, as well as lower intakes of fiber, polyunsaturated fatty acids, micronutrients, and bioactive compounds with antioxidant properties [76,77,78]. Similarly, lower adherence to the MIND diet or healthy dietary patterns may reflect more limited consumption of foods rich in fiber, polyunsaturated fatty acids, vitamins, minerals, and bioactive compounds with antioxidant properties [79]. Experimental and broader neurocognitive literature has suggested that diets high in fat and refined sugar may potentially affect metabolic regulation, hippocampal function, BDNF levels, and neuronal plasticity [80,81]. However, a substantial proportion of this evidence has been derived from experimental models, and the relevance of these proposed pathways to childhood ADHD has not yet been directly established. Moreover, because these processes were not assessed in the present study, they should be regarded as conceptual possibilities for interpreting the findings rather than as mechanisms supported by our results.

Furthermore, the observed dietary characteristics may reflect not only child-specific factors associated with ADHD but also parental dietary habits and feeding practices, food availability at home, and broader familial, socioeconomic, and cultural conditions. Accordingly, the possibility of unmeasured or residual confounding cannot be excluded.

Differences in reward processing and inhibitory control associated with ADHD may contribute to greater consumption of highly processed foods [66]. As many of these products are formulated to be highly palatable and easily consumed, such characteristics could plausibly increase their appeal. However, these processes were not directly assessed in the present study, and this explanation should therefore be regarded as a conceptual possibility rather than a mechanism supported by our findings.

Although higher desire to drink and sQ-HPF scores were observed in the present study, these findings do not indicate which types of beverages were preferred. The higher consumption of water and ayran, as well as energy drinks, among children with ADHD makes it difficult to explain the findings solely in terms of reward-driven beverage seeking. Whether these differences reflect physiological thirst, a general preference for liquids, sensory preferences, or a combination of these factors cannot be determined from self-reported intake data in the absence of objective indicators of hydration.

Given the observational design, ADHD-related characteristics may have shaped meal patterns, dietary variety, and preferences for easily consumed products over time. Indeed, in a population-based cohort study using bidirectional analyses, ADHD symptoms at age six predicted lower diet quality at age eight, whereas diet quality at age eight did not predict ADHD symptoms at age ten [82]. This finding supports the possibility that ADHD-related characteristics may precede and shape subsequent dietary quality, although it does not establish the direction of the association observed in the present study.

### 4.4. Strengths and Limitations of This Study

A major strength of the present study was the inclusion of an age- and sex-matched (within ±6 months), carefully characterized sample of children with newly diagnosed, treatment-naïve ADHD and without major neurodevelopmental, psychiatric, neurological, or chronic comorbidities. This design reduced the potential influence of treatment and co-occurring conditions on eating behaviors and dietary characteristics. The multidimensional assessment of eating behaviors, dietary indices, highly processed food consumption, and empirically derived dietary patterns using complementary instruments, together with anthropometric measurements and nutrition-related biochemical indicators was another important strength. Furthermore, the matched design was accounted for in the group comparisons and conditional logistic regression analyses, while the expanded sensitivity models incorporated additional socioeconomic and family-related covariates.

Several limitations should nevertheless be considered. First, the observational case–control design does not establish temporal ordering or causality among eating behaviors, dietary characteristics, and ADHD. Eating behaviors and habitual dietary intake were assessed primarily through child and parent reports and may therefore be affected by recall error, social desirability bias, and measurement error. Moreover, the researcher conducting the in-person assessments was aware of group assignment. Although standardized procedures were followed, the absence of assessor blinding may have introduced interviewer bias in the subjective, interview-based assessments. The study was conducted at a single center using a relatively small and highly selected clinical sample; consequently, the findings may not be generalizable. The findings were not stratified by ADHD presentation and symptom severity, limiting the evaluation of clinical heterogeneity within the ADHD group. The relatively small number of matched pairs may have limited estimate precision and model stability, particularly in the median-dichotomized analyses and the expanded sensitivity models including additional covariates. Accordingly, estimates with wide confidence intervals should be interpreted cautiously. The Turkish version of the sQ-HPF was validated in adults rather than in a pediatric population; therefore, its use in Turkish children represents a measurement-related limitation that should be considered when interpreting the sQ-HPF findings. Moreover, the use of sample-specific tertiles to score the MIND Diet Index limits the external interpretability of the absolute scores and their direct comparability across populations. The use of broad and nutritionally heterogeneous food groups in the PCA may have obscured food-specific associations and limits the external interpretability of the derived patterns. Objective markers of hydration and thirst were not assessed, preventing determination of whether the observed beverage-oriented behaviors reflected physiological thirst, sensory preferences, reward-related beverage seeking or other mechanisms. Finally, although the primary and sensitivity models accounted for selected sociodemographic characteristics, residual confounding cannot be excluded, and the exposure-specific estimates should not be interpreted as mutually independent or causal effects.

## 5. Conclusions

In this exploratory age- and sex-matched case–control study, higher desire-to-drink scores were associated with higher odds of ADHD. Children with ADHD also had lower MIND Diet Index scores and higher continuous sQ-HPF scores. In separate exposure-specific analyses, their associations with ADHD odds were inverse and positive, respectively. These findings should be interpreted in the context of a selected sample of newly diagnosed, treatment-naïve children without major comorbidities. These exploratory findings should be evaluated in larger, independent, multicenter prospective studies incorporating repeated and, where feasible, objective dietary assessments and detailed characterization of ADHD presentations and symptom severity to clarify temporal direction and potential underlying processes.

## Figures and Tables

**Figure 1:**
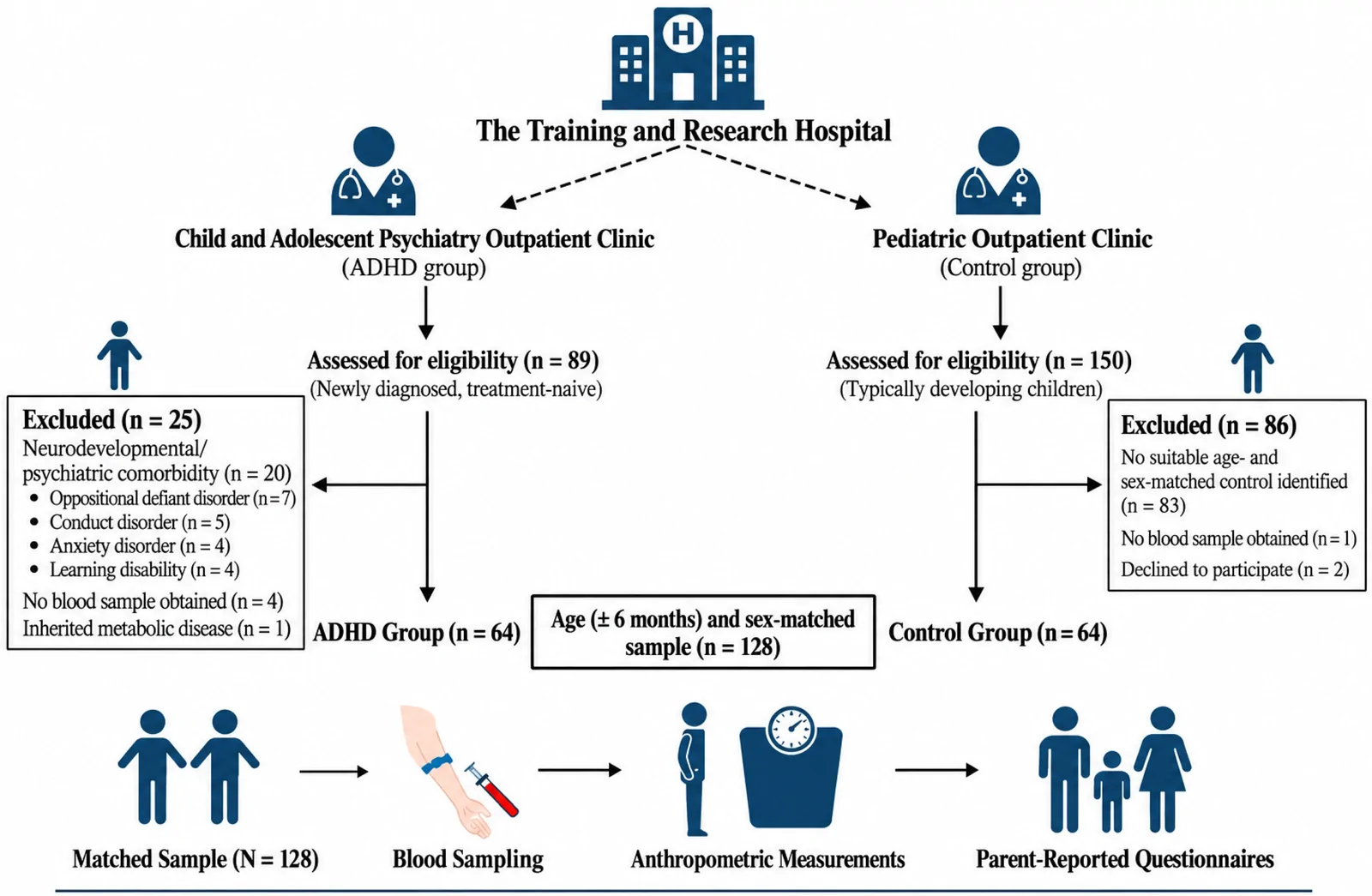
Sample and data collection process.

**Figure 2:**
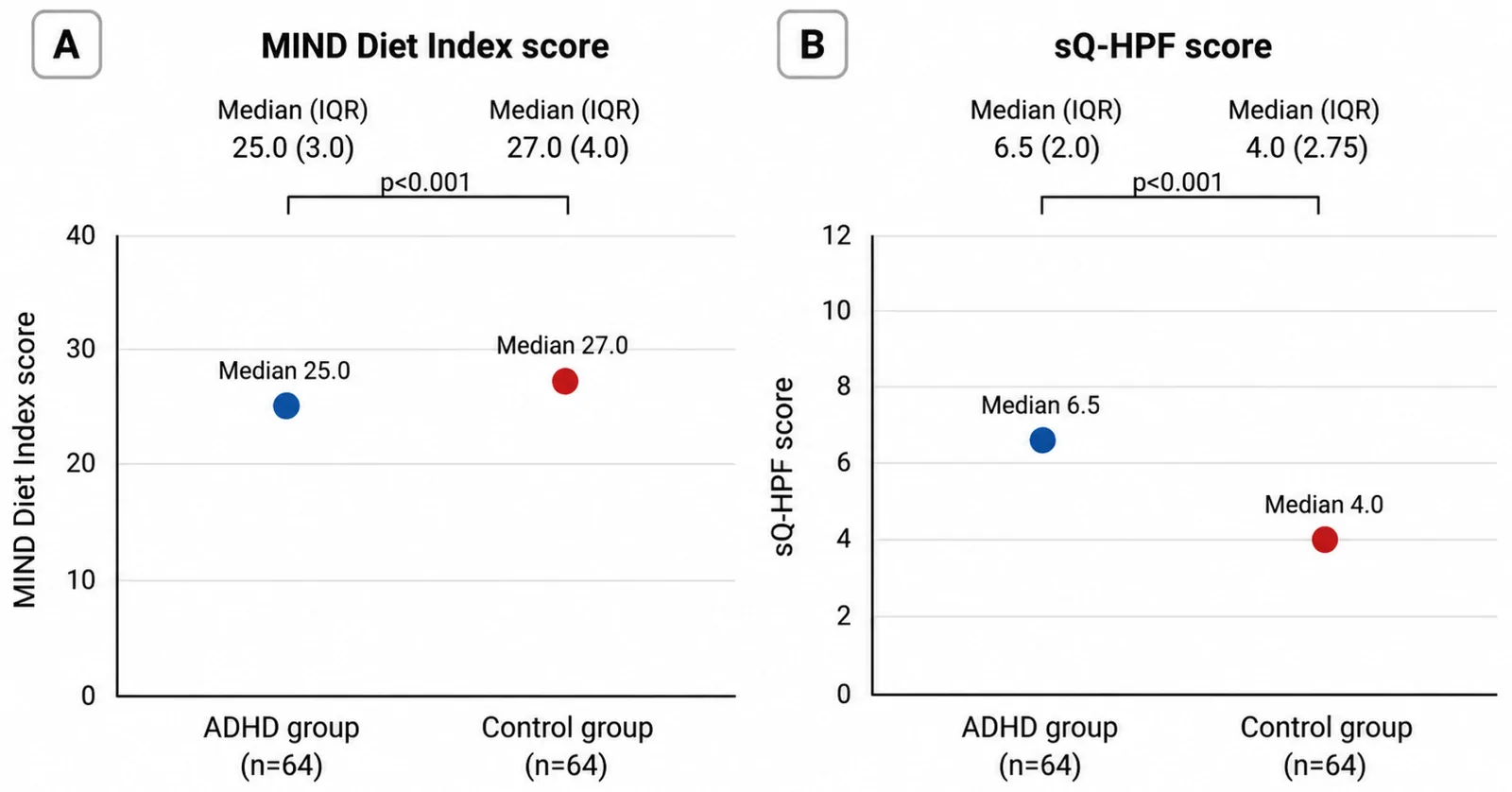
MIND Diet Index (**A**) and sQ-HPF (**B**) scores in children with ADHD and age- and sex-matched healthy controls. Points represent median values, and the corresponding interquartile ranges (IQRs) are reported above each group. Between-group comparisons were performed using the Wilcoxon signed-rank test.

**Figure 3:**
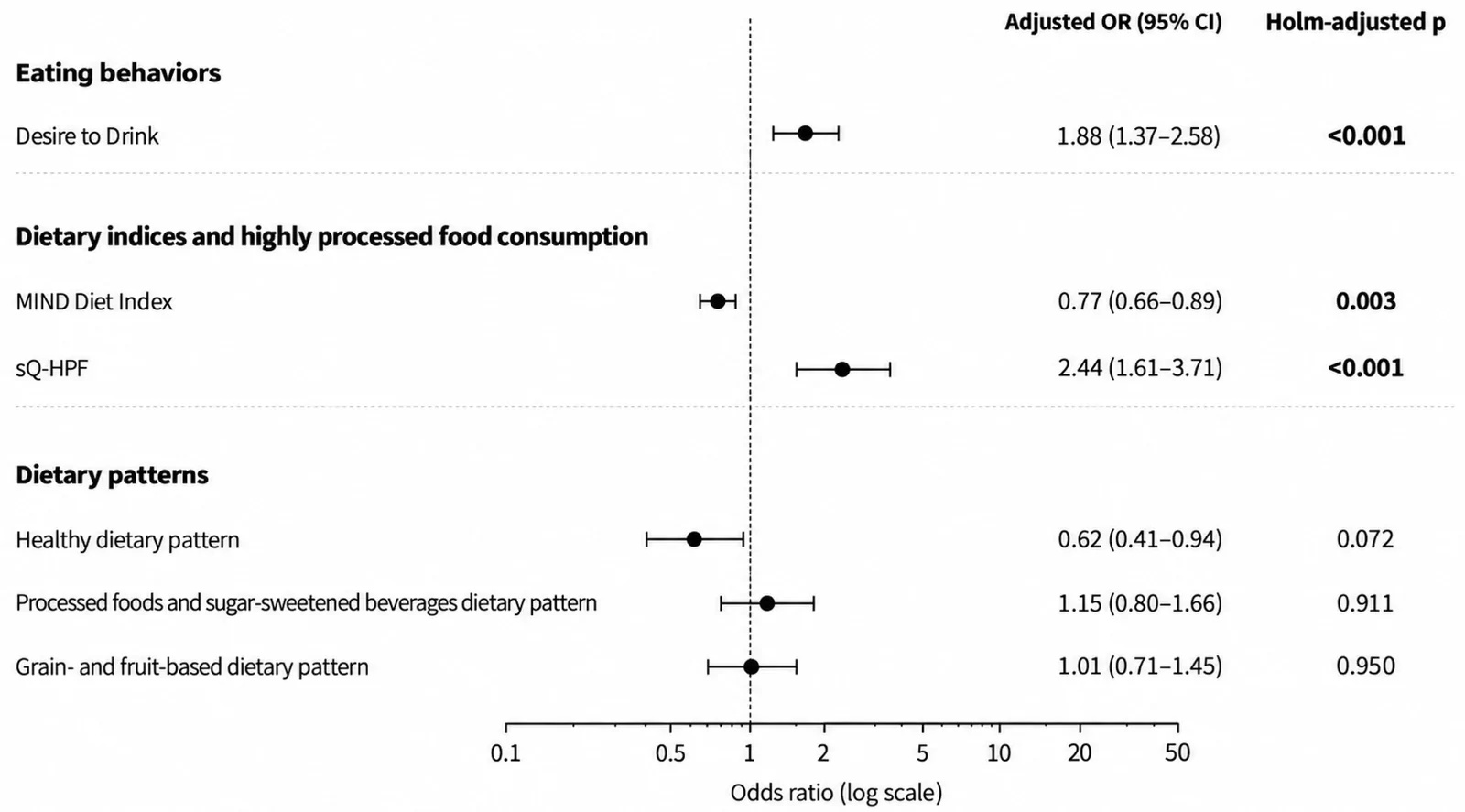
Forest plot of the associations between eating-behavior and dietary exposures and ADHD status in the primary adjusted model (Model 2: adjusted for maternal education). Points represent adjusted matched odds ratios (ORs), and horizontal lines represent 95% confidence intervals (CIs). The vertical dashed line indicates an OR of 1.00. Holm correction was applied across the six continuous exposure analyses, and the corresponding Holm-adjusted *p* values are shown; statistically significant adjusted *p* values are presented in bold. ORs > 1 indicate higher odds of ADHD, whereas ORs < 1 indicate lower odds.

**Table 1:** Demographic, socioeconomic, lifestyle, anthropometric and biochemical characteristics of children with ADHD and age- and sex-matched healthy controls.

Variables	ADHD (n = 64)	Control (n = 64)	*p* Value
**Demographic and socioeconomic characteristics**			
**Age (years), mean ± SD**	8.90 ± 2.46	8.98 ± 2.49	—
**Sex, n (%)**			
Male	46 (71.9)	46 (71.9)	—
Female	18 (28.1)	18 (28.1)	—
**Parental marital status, n (%)**			
Married	56 (87.5)	63 (98.4)	**0.039** †
Divorced/Widowed	8 (12.5)	1 (1.6)	
**Maternal education, n (%)**			
Low (illiterate/primary school)	11 (17.2)	16 (25.0)	0.510 ‡
Medium (secondary/high school)	40 (62.5)	38 (59.4)	
High (university or above)	13 (20.3)	10 (15.6)	
**Family Affluence Scale-II**			
FAS-II total score, median (Q1–Q3)	4 (2–5)	4 (3–4)	0.641 *
Low n (%)	19 (29.7)	15 (23.4)	**0.028** ‡
Medium n (%)	35 (54.7)	48 (75.0)	
High n (%)	10 (15.6)	1 (1.6)	
**Lifestyle characteristics**			
Daily screen time median, (Q1–Q3), h/day	3 (2–6.43)	3 (2–4.29)	0.222 *
Daily sleep duration, median (Q1–Q3), h/day	9 (8.5–10)	9 (8–9)	0.074 *
**Regular physical activity, n (%)**			
Yes	12 (18.8)	9 (14.1)	0.607 †
No	52 (81.2)	55 (85.9)	
**Outdoor play frequency, n (%)**			
Low (never or 1–2 days/week)	15 (23.4)	28 (43.8)	**0.014** ‡
Medium (3–4 days/week)	12 (18.8)	17 (26.6)	
High (5–6 days/week or every day)	37 (57.8)	19 (29.7)	
Anthropometric characteristics *			
Height (cm)	132.0 (124.3–147.0)	131.0 (125.0–145.5)	0.507
Weight (kg)	30.8 (25.0–43.2)	28.9 (24.1–41.0)	0.121
BMI-for-age z-score (BAZ)	0.59 (0.12–1.70)	0.51 (−0.65–1.50)	0.126
Waist-to-height ratio	0.454 (0.429–0.498)	0.450 (0.426–0.489)	0.301
Body fat percentage (%)	22.1 (17.6–28.9)	20.9 (16.1–27.3)	0.242
Mid-upper arm circumference (cm)	19.0 (17.0–21.8)	18.0 (17.0–22.0)	**0.015**
Biochemical characteristics *			
Potassium (mmol/L)	4.44 (4.23–4.57)	4.51 (4.34–4.88)	0.156
Calcium (mg/dL)	9.80 (9.60–10.03)	9.80 (9.60–9.97)	0.517
Magnesium (mg/dL)	2.04 (1.95–2.17)	2.05 (1.97–2.12)	0.817
Serum iron (µg/dL)	65.1 (46.2–96.7)	64.2 (49.6–94.8)	0.779
Ferritin (ng/mL)	20.5 (14.4–32.0)	26.0 (18.0–42.3)	**0.006**
Total iron-binding capacity (µg/dL)	349.5 (319.6–379.4)	327.5 (297.0–357.9)	**0.023**
Vitamin B12 (pg/mL)	383.5 (301.1–515.9)	348.0 (292.0–430.5)	0.243
Folate (ng/mL)	9.14 (6.67–13.68)	9.79 (7.66–13.11)	0.262

Values are presented as mean ± SD, median (Q1–Q3), or n (%), as appropriate. Age and sex were matching variables and were therefore not subjected to significance testing. Statistically significant *p* values are shown in bold. * Wilcoxon signed-rank test; † McNemar’s exact test; ‡ McNemar–Bowker test. Q1, first quartile; Q3, third quartile; SD, standard deviation.

**Table 2:** Eating behavior scores in children with ADHD and age- and sex-matched healthy controls.

Variable	ADHD (n = 64) Median (Q1–Q3)	Control (n = 64) Median (Q1–Q3)	Z	*p* Value
**Children’s Eating Behaviour Questionnaire (CEBQ)**				
**Food-avoidant eating behaviors**				
Satiety responsiveness	1.50 (1.00–3.18)	1.86 (1.00–3.32)	−0.341	0.733
Slowness in eating	1.00 (1.00–2.50)	1.00 (1.00–2.69)	−0.070	0.944
Food fussiness	3.00 (2.00–4.00)	3.00 (2.00–4.00)	−0.272	0.785
Emotional undereating	1.00 (1.00–1.00)	1.00 (1.00–1.00)	−0.649	0.516
**Food-approach eating behaviors**				
Food responsiveness	1.70 (1.00–2.75)	1.00 (1.00–2.10)	−1.493	0.135
Enjoyment of food	3.10 (2.10–3.80)	3.10 (2.20–3.60)	−0.023	0.981
Desire to drink	4.00 (2.00–4.67)	2.00 (1.00–3.00)	−4.467	**<0.001**
Emotional overeating	1.00 (1.00–1.00)	1.00 (1.00–1.00)	−0.362	0.717
**Behavioral Pediatrics Feeding Assessment Scale (BPFAS)**				
Food selectivity	17 (15–21)	17 (15–21)	−0.150	0.880
Early food refusal	8 (5–12)	9 (5–11)	−0.300	0.767
Early texture-related food refusal	5 (5–5)	5 (5–5)	−1.610	0.106
Late food refusal	19 (16–24)	15 (13–18)	−4.080	**<0.001**

CEBQ subscale scores were calculated as the mean of the constituent item scores, whereas BPFAS subscale scores were calculated by summing the constituent item scores after reverse scoring the positively worded items. Values are presented as median (Q1–Q3), and paired comparisons were performed using the Wilcoxon signed-rank test. A Bonferroni correction was applied separately within each instrument, resulting in adjusted significance thresholds of *p* < 0.00625 for the eight CEBQ subscales and *p* < 0.0125 for the four BPFAS subscales. Results remaining statistically significant after correction are shown in bold. Q1, first quartile; Q3, third quartile; Z, standardized Wilcoxon signed-rank test statistic.

**Table 3:** Associations of eating behaviors, dietary indices, highly processed food consumption, and dietary patterns with ADHD.

Variable	Model 1 ^1^			Model 2 ^2^			Model 3 ^3^		
	OR	95% CI	Nominal P Holm-Adjusted *p*	OR	95% CI	Nominal P Holm-Adjusted *p*	OR	95% CI	Nominal P Holm-Adjusted *p*
**Eating behaviors**									
**CEBQ Desire to drink score**									
Continuous	1.82	(1.35–2.45)	<0.001 **<0.001**	1.88	(1.37–2.58)	<0.001 **<0.001**	1.87	(1.34–2.60)	<0.001 **0.001**
At or below the median	Ref			Ref			Ref		
Above the median	4.83	(2.29–10.20)	<0.001 **<0.001**	4.50	(1.96–10.32)	<0.001 **0.002**	4.39	(1.85–10.45)	0.001 **0.004**
**Dietary indices and highly processed food consumption**									
**MIND Diet Index score**									
Continuous	0.77	(0.66–0.90)	0.001 **0.006**	0.77	(0.66–0.89)	0.001 **0.003**	0.74	(0.61–0.88)	0.001 **0.004**
At or below the median	Ref			Ref			Ref		
Above the median	0.36	(0.17–0.74)	0.005 **0.020**	0.33	(0.15–0.69)	0.003 **0.014**	0.26	(0.11–0.62)	0.002 **0.009**
**sQ-HPF score**									
Continuous	2.23	(1.54–3.24)	<0.001 **<0.001**	2.44	(1.61–3.71)	<0.001 **<0.001**	2.42	(1.57–3.73)	<0.001 **<0.001**
At or below the median	Ref			Ref			Ref		
Above the median	5.67	(2.38–13.50)	<0.001 **<0.001**	5.86	(2.41–14.24)	<0.001 **<0.001**	5.93	(2.32–15.13)	<0.001 **0.001**
**Dietary patterns**									
**Healthy dietary pattern**									
Continuous	0.64	(0.43–0.95)	0.027 0.081	0.62	(0.41–0.94)	0.024 0.072	0.54	(0.33–0.88)	0.014 **0.041**
At or below the median	Ref			Ref			Ref		
Above the median	0.68	(0.42–1.13)	0.136 0.408	0.46	(0.22–0.95)	0.036 0.109	0.40	(0.17–0.92)	0.030 0.091
**Processed foods and sugar-sweetened beverages dietary pattern**									
Continuous	1.09	(0.77–1.54)	0.636 1.000	1.15	(0.80–1.66)	0.455 0.911	1.11	(0.75–1.65)	0.592 1.000
At or below the median	Ref			Ref			Ref		
Above the median	0.63	(0.28–1.38)	0.244 0.488	0.68	(0.29–1.62)	0.390 0.445	0.57	(0.23–1.44)	0.238 0.257
**Grain- and fruit-based dietary pattern**									
Continuous	1.03	(0.72–1.46)	0.893 1.000	1.01	(0.71–1.45)	0.948 0.950	0.94	(0.65–1.37)	0.749 1.000
At or below the median	Ref			Ref			Ref		
Above the median	0.67	(0.32–1.38)	0.277 0.488	0.63	(0.30–1.33)	0.223 0.445	0.54	(0.24–1.20)	0.129 0.257

Each exposure was examined separately using conditional logistic regression accounting for matching. Holm correction was applied separately to the six continuous and six median-dichotomized analyses within each model. Bold indicates a Holm-adjusted *p* value < 0.05. ^1^ Model 1 was the unadjusted exposure-specific matched model. ^2^ Model 2, designated as the primary adjusted model, included maternal education. ^3^ Model 3 was an expanded sensitivity model that included maternal education, the FAS-II total score and parental marital status.

## Data Availability

The data presented in this study are available on request from the corresponding author due to ethical restrictions and participant confidentiality.

## Supplementary Materials

The following supporting information can be downloaded at https://www.mdpi.com/article/10.3390/nu18193176/s1: Table S1: Food group classification used for dietary pattern analysis; Table S2: Item-level comparison of the BPFAS late-food-refusal subscale between children with ADHD and age- and sex-matched healthy controls; Table S3: Rotated factor loadings for the three dietary patterns identified by principal component analysis.
