# Supplementary material for: Eating Behaviors, Dietary Patterns and Diet Quality in Newly Diagnosed, Treatment-Naïve Children with ADHD: An Age- and Sex-Matched Case–Control Study
Source: Nutrients. 2026 Sep 26;18(19):3176. doi: 10.3390/nu18193176 (PMC13666592; doi:10.3390/nu18193176)
Supplement: Supplementary file 1 [file nutrients-18-03176-s001.zip › nutrients-4550137-supplementary.pdf]

Supplementary material

Table S1. Food group classification used for dietary pattern analysis

Food groups	food items included
**Vegetables**	Cabbage, cauliflower, carrots, tomatoes, leafy green vegetables (spinach, lettuce, cucumber), eggplant, artichoke, peas, green beans, peppers, zucchini, okra, chard, purslane, parsley, radish, basil, green onions
**Fruits**	Pears, quinces, apricots, cherries, apples, grapes, sour cherries, bananas, melons, watermelons, oranges, strawberries, peaches, nectarines, mandarins, mulberries, plums, figs, pomegranates, prunes
**Animal-source foods**	Red meat, meatballs and kebabs, chicken, turkey, eggs, fish, offal, seafood
**Grains**	Whole grain breads, white bread, bulgur, rice, pasta, toast, flatbread
**Legumes**	Lentils, beans, chickpeas, kidney beans
**Nuts, seeds and olives**	Peanuts, almonds, pistachios, hazelnuts, roasted seeds, walnuts, olives, olive oil
**Dairy products**	Milk, yogurt, buttermilk, kefir, cheeses (cheddar, curd, white cheese)
**Processed and ultra-processed foods**	Ready-made pastries, potato chips, popcorn, chocolate, cookies, cakes, candies, halva, syrupy desserts, puddings, ice cream, processed meats (salami, smoked and sausage), breakfast cereals, biscuits, sauces (ketchup, mayonnaise, etc.)
**Sugar-sweetened beverages**	Cola, sodas, packaged fruit juice, energy drinks, iced tea, taster's choice, sugary tea, sugary coffee

Food items were grouped according to their nutritional characteristics and degree of processing based on previous dietary pattern studies.

Table S2. Item-level comparison of the BPFAS late-food-refusal subscale between children with ADHD and age- and sex-matched healthy controls

Late-food-refusal item	ADHD (n = 64) Median (Q1–Q3)	Control (n = 64) Median (Q1–Q3)	Z	*p* value
Drinks milk	5 (4–5)	4 (3–5)	−1.612	0.107
Eats snacks between meals	4 (3–4)	4 (3–5)	−0.894	0.371
Leaves the table during meals	2 (1–2.5)	1 (1–1)	−1.487	0.137
Delays eating by talking	2 (1–4)	2 (1–2)	−2.326	0.020
Prefers liquids instead of solid foods	3.5 (2–4)	1 (1–1)	−4.854	**<0.001**
Refuses to eat at mealtime but asks for food after the meal	2 (1–2)	2 (1–2)	−1.221	0.222
Argues about what he/she will or will not eat	3 (2–4)	1 (1–3)	−2.503	0.012

Values are presented as median (Q1–Q3) of item scores ranging from 1 to 5. Paired comparisons were performed using the Wilcoxon signed-rank test. Unadjusted p values are presented. A Bonferroni correction was applied across the seven item-level comparisons, resulting in an adjusted significance threshold of $p < 0.0071$. Results that remained statistically significant after Bonferroni correction are shown in bold. BPFAS, Behavioral Pediatrics Feeding Assessment Scale; Q1, first quartile; Q3, third quartile; Z, standardized Wilcoxon signed-rank test statistic.

Table S3. Rotated factor loadings for the three dietary patterns identified by principal component analysis

Food Groups	Healthy dietary pattern	Processed foods and sugar-sweetened beverages dietary pattern	Grain and fruit-based dietary pattern
**Vegetables**	**0.706**	-0.088	0.038
**Animal-source foods**	**0.705**	-0.127	0.276
**Dairy products**	**0.650**	0.240	0.070
**Legumes**	**0.457**	0.232	-0.022
**Nuts, seeds and olives**	**0.504**	0.141	-0.193
**Processed and ultra-processed foods**	-0.029	**0.799**	0.227
**Sugar-sweetened beverages**	0.067	**0.771**	-0.105
**Fruits**	0.085	-0.113	**0.784**
**Grains**	-0.004	0.215	**0.747**

The three dietary patterns explained 53.9% of the total variance. Only factor loadings ≥0.30 are shown in bold.
